# Genetic Factors in Parkinson’s Disease

**DOI:** 10.2174/138920291408140206113810

**Published:** 2013-12

**Authors:** Jolanta Dorszewska

Parkinson's disease was discovered almost 200 years ago. Despite significant progress in the diagnosis and treatment of Parkinson's disease, it remains an incurable disease with an observed constantly increasing mortality.

The exact etiology of Parkinson’s disease remains unknown. Currently it is believed that Parkinson’s disease occurs due to an interaction between environmental and genetic factors. Recently, there have been many significant developments in understanding the genetic mechanisms involved in Parkinson’s disease.

The first discoveries in the genetics of Parkinson's disease appeared in the 1980s. The main pathological protein of this degenerative disease, alpha-synuclein, was discovered in 1988.

A mutation first described in the Italian Contrusi family’s *SNCA* gene, encoding the protein alpha-synuclein, was described in 1990. At the same time, it was first described as a mutation in Parkinson's disease. However, in 1998 the first described mutation in the *PRKN* gene encoding the Parkin protein was found. The turn of the XXI has seen a breakthrough in the study of the genetic determinant of Parkinson's disease. A number of genes associated with the onset of familial Parkinson's disease and/or responsible of labeled PARK have been described.

It appears that familial Parkinson’s disease is triggered by several very infrequent mutations, with age dependent, robust penetration, inherited in autosomal recessive or dominant way, in accordance with Mendelian. The presence of these mutations is sufficient factor to onset of the disease.

On the other hand, the sporadic Parkinson’s disease is a multifactorial, extremely complex condition, where the genetic factors play rather modest role, however when combined together with other genetic changes and environmental factors are potent enough to inflict the disease as a result of an additive effect.

The data obtained in past decade has linked five genes (*SNCA, LRRK2, PRKN, DJ-1* and *PINK1*) to the familial Parkinson’s disease. The recent discovery of additional 6 loci involved in the sporadic disease (*MAPT, SNCA, HLA-DRB5, BST1, GAK* and *LRRK2*) has been made through genome wide association studies (GWAS) performed on various populations.

It seems that genetic analysis of PARK genes in Parkinson’s disease may improve the diagnostic and prognostic process in this disease and can contribute to more effective pharmacotherapy.

